# Novel Starch-PVA Polymer for Microparticle Preparation and Optimization Using Factorial Design Study

**DOI:** 10.1155/2015/261476

**Published:** 2015-01-12

**Authors:** Helen Chattopadhyay, Amit Kumar De, Sriparna Datta

**Affiliations:** Department of Chemical Technology, University of Calcutta, 92 A.P.C. Road, Kolkata 700 009, India

## Abstract

The aim of our present work was to optimize the ratio of a very novel polymer, starch-polyvinyl alcohol (PVA), for controlled delivery of Ornidazole. Polymer-coated drug microparticles were prepared by emulsion method. Microscopic study, scanning electron microscopic study, and atomic force microscopic study revealed that the microparticles were within 10 micrometers of size with smooth spherical shape. The Fourier transform infrared spectroscopy showed absence of drug polymer interaction. A statistical 3^2^ full factorial design was used to study the effect of different concentration of starch and PVA on the drug release profile. The three-dimensional plots gave us an idea about the contribution of each factor on the release kinetics. Hence this novel polymer of starch and polyvinyl alcohol can be utilized for control release of the drug from a targeted delivery device.

## 1. Introduction

To date, oral delivery is still the preferred route of drug administration, especially for chronic patients where repeated administration is required. Oral administration offers less pain, greater convenience for patients, higher compliance, and reduced risk of cross-infection and needle stick injuries [1, 2].

Controlled drug delivery technology using natural biodegradable polymers as carrier represents one of the most rapidly advancing areas of science. Poly(esters), polyacrylate, natural gums, and polysaccharides are used for controlled release drug delivery device because these are biodegradable, nontoxic, freely available, and less expensive polymeric materials for use in controlled drug delivery system [3–5]. However, these materials have certain drawbacks, like uncontrolled rate of hydration, unusual thickening, drop in viscosity on storage, and microbial contamination and they require functionalization/modification to overcome these problems. The use of natural gums such as xanthan gum [6], guar gum [7], gum Arabic [8], and gellan gum [9, 10] and polysaccharides such as chitosan, pectin, chondroitin sulphate, cyclodextrins, and dextrose in drug delivery devices is well documented.

Native potato starch is a compound of two polysaccharides: branched amylopectin, 78%, and linear amylose, 22%. Different starch derivatives are used for drug coating as mentioned in literature like the film-forming ability of starch acetate (SA) and the effect of commonly used plasticizers on the physical properties of SA films was evaluated [11]. The products of starch are mostly brittle and water soluble. These properties of starch can be improved by blending with synthetic polymers [12].

PVA is attractive for its excellent film forming, oil-grease-solvent resistance, flexibility, nontoxicity, emulsifying, and adhesiveness, as well as high oxygen and aroma barrier properties. Starch-PVA films can also be used in biomedical and clinical field as control release drug carrier and biomembrane.

In our work we have used a novel starch-PVA polymer as drug carrier as it is easily degraded in the system. When PVA is linked with potato starch, the polymer becomes economically more viable compared to PVA alone. Process ability [13], mechanical properties [14], and biodegradability of starch-PVA mixtures have been studied in detail [15–17]. We found that, during biodegradation, starch degraded first followed by the amorphous region of the PVA [18]. Unfortunately detailed literature survey revealed no work using this starch-PVA polymer for control release of a drug, till today. Hence we attempted to prepare microparticle of Ornidazole as a model drug with novel starch-PVA polymer having optimum release characteristics.

The drug Ornidazole [1-(2-hydroxy-3-chloropropyl)-2-methyl-5-nitro-imidazole] is a synthetic derivative of 5-nitroimidazole and has antiprotozoan and antibacterial properties against anaerobic bacteria* H. pylori*. Ornidazole is the preferred drug used in the treatment for severe hepatic and intestinal amoebiasis, giardiasis, and trichomoniasis of the urogenital tract, bacterial vaginosis, anaerobic dental infections and gastric surgery, the management of induced duodenal ulcers, inflammatory bowel disease (IBD), and Crohn's disease (CD) [19]. Due to complete and early absorption after oral administration and short biological half-life, Ornidazole becomes unavailable for local action in later part of the GI tract and colon. To overcome this problem in our study we have used Ornidazole in a controlled release delivery device with starch-PVA polymer.

To bind the drug with the polymers different types of crosslinking agents like glutaraldehyde [20], sulphuric acid, sodium tripolyphosphate, citric acid [21], and calcium chloride [22] have been used widely which control the drug release profile. In our study we have used glutaraldehyde as the crosslinking agent. The crosslinking starts within 5 min after the addition of glutaraldehyde and with increasing time crosslinking increases. We have given 4 hrs for total crosslinking process. We have used acidic medium by adding concentrated orthophosphoric acid to get the good crosslinking action of glutaraldehyde.

We have used statistical experimental design techniques like factorial design and response surface methodology as useful tools [23] to optimize the ratio of novel polymers for control release of Ornidazole from the polymer-coated microparticle.

## 2. Materials and Methods

Ornidazole was obtained from Dey's Medical Stores (Mfg) Ltd., Kolkata. The starch-polyvinyl alcohol blend was obtained from Polymer Science & Technology Department, University of Calcutta. PVA (LOBA Chemie Pvt., Ltd.), starch (Merck.), heavy liquid paraffin (Merck), Span 20 (s.d. Chem Pharma Ltd.), glutaraldehyde 25% (Merck), orthophosphoric acid (Merck), and n-hexane (Merck) were all purchased from the local chemical suppliers. All the chemicals and reagents were of analytical reagent grade. We used double distilled water for all the experiments.

### 2.1. Preparation of Microparticles

Starch-PVA particle containing Ornidazole was prepared by emulsion method [24]. A calculated amount of starch and PVA was added to distilled water to prepare 100 mL polymer solution and the mixture was gelatinized at 80°C for 120 min. Aqueous solution of Ornidazole was prepared to which required amount of heavy liquid paraffin and 1% Span 20 (as emulsifier) were added and separately mixed using magnetic stirrer. Drug and polymer were added dropwise to the oil phase at stirring condition which was followed by sonication and homogenization. Required amount of orthophosphoric acid was added to make the solution acidic. Glutaraldehyde, the cross linker, was added to the emulsion at stirring condition. After 4 hours of stirring the emulsion was centrifuged for 15 minutes at 10,000 RPM (REMI R24). The oil phase, aqueous phase, and the product were finally separated. The product was washed with n-hexane to remove the residual oil at the surface and was stored in desiccators for further characterization and analysis. The aqueous phase was collected, diluted, and subjected to UV analysis in Shimadzu UV Visible Spectrophotometer (Model number UV2550) for the residual drug in the solution. The compositions of different formulations were presented in Table 1.

### 2.2. Percentage Drug Loading

The stock solution of the drug was prepared having strength of 1 mg/mL. It was diluted 100 times, UV absorbance was measured at 312 nm, and the exact concentration of the drug in the solution was determined from the calibration curve using equation, that is, *y* = 0.037*x* + 0.009; *R* ^2^ = 0.992, where *y* = absorbance and *x* = concentration in *μ*g/mL. The aqueous phase of every formulation after preparation of microparticles was diluted and absorbance was measured at the same wavelength for determination of the concentration of the residual drug in the aqueous phase. From the difference between the initial concentration of the drug in solution and residual drug concentration in aqueous phase, percentage drug loading in microparticles was calculated. The data were presented in Table 2.

### 2.3. In Vitro Drug Release Study

In vitro drug release study was performed for all formulations F1 to F9 in a USP type I dissolution tester (Electrolab TDL-08L, India) at a rotational speed of 100 rpm and temperature of 37 ± 0.5°C. The test was performed using 900 mL of 0.1 N HCl at 37 ± 0.5°C and 100 rpm for first 2 h. Then the dissolution was continued with pH 6.8 phosphate buffer [25]. After definite time intervals, 5 mL aliquot was withdrawn and absorbance was noted with this sample at 312 nm and each time 5 mL fresh buffer solution was replenished. This study was continued up to 48 hours. The results were presented in factorial design layout table.

### 2.4. Optimization Using 3^2^ Factorial Design

A 3^2^ (three level two factors) full-fledged factorial design was applied to optimize the two independent variables, namely, the amount of starch (*X*_1_) and the amount of PVA (*X*_2_). The drug release in pH = 6.8 phosphate buffer medium was monitored first at 30 min and then at every hour. The releases at 180 min (3 hrs), 1440 min (24 hrs), and 2880 min (48 hrs) were analyzed as response parameters by the factorial design studies. Results were expressed as the second order polynomial equation:

(1) $$\begin{array}{c} Y_{i}=b_{0}+b_{1}X_{1}+b_{2}X_{2}+b_{12}X_{1}X_{2}+b_{11}X_{1}^{2}+b_{22}X_{2}^{2}, \end{array}$$

where *b*_0_ denoted the arithmetic mean response for nine runs and *b*_*i*_ (*i* = 1,2) denoted the estimated coefficient for the factors, *X*_*i*_ (*i* = 1,2). *X*_*i*_ (*i* = 1,2) denoted the effect of changing one factor at a time from its lowest to highest level. The interaction terms *X*_1_ *X*_2_ denoted the effect when both factors were changed simultaneously. The polynomial terms *X*_*i*_^2^ (*i* = 1,2) were used to explain nonlinearity. *Y* was the measured response parameter in each experiment. The coefficients corresponding to the linear effects *b*_1_ and *b*_2_, interaction *b*_12_, and the quadratic effects, *b*_11_ and *b*_22_, were determined from the experimental results.

### 2.5. Characterisation of Ornidazole Loaded Starch-PVA Microparticles

#### 2.5.1. Microscopic Study

The microparticles were examined under microscope and size was measured by the software Motic Image Plus 2.0. The required amount of the product was dispersed in isopropyl alcohol solution and different dilutions were made. Glass slides were prepared by adding drops of different dilutions and placed under the microscope for analysis. The particles were measured at 40 times magnification.

#### 2.5.2. Fourier Transform Infrared (FTIR) Spectroscopic Study

The infrared spectra of Ornidazole, starch, PVA polymer, and drug loaded starch-PVA microparticles were obtained by pressed KBr pellet technique using Jasco 670Plus FTIR Spectrophotometer.

#### 2.5.3. Atomic Force Microscopic (AFM) Study

The surface morphology of the polymer-coated Ornidazole microparticles was further analysed using conventional tapping mode of AFM (Nanoscope IIIa controller with Multimode AFM, Veeco, Cambridge, UK). The sample was prepared by adding drops of different dilutions on the clean glass cover slip. Samples were mounted on carbon sticky tabs and imaged in air using tapping mode tips. This study showed the distribution of Ornidazole within the polymer matrix.

#### 2.5.4. Scanning Electron Microscopic (SEM) Study

The surface morphology and size of starch-PVA polymer-coated Ornidazole microparticles were further visualized under SEM, Ion Sputter Hitachi S-3400N, Japan, at 15 kV.

### 2.6. Statistical Analysis

Statistical analysis of the drug release studies was carried out using the SigmaPlot Software (Version 8.02 SPSS Inc., USA).

## 3. Results and Discussion

### 3.1. Percent Drug Loading

The results showed that the percent drug loading was 43% for F2 and 41% for F3 and F9, whereas % drug loading decreased below 40% for all other formulations. The results were mentioned in Table 2.

### 3.2. In Vitro Drug Release Study

**Figure 1:**
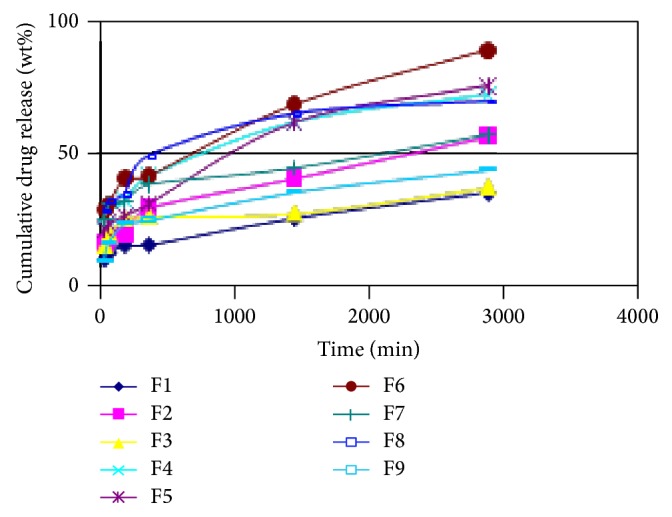
Cumulative drug release study for formulations F1 to F9 in pH = 6.8 phosphate buffer medium.

There was no drug release observed in 0.1 N HCl medium from all the formulations. In pH = 6.8 phosphate buffer medium drug release from all formulations F1 to F9 showed a slow release pattern starting from 30 minutes to 2880 minutes (48 hrs) (Figure 1). For the first set of formulations F1, F2, and F3, where starch was maximum, drug release was minimum for the entire time period. From the second set F4, F5, and F6 where the amount of starch was medium, the drug release was very high. But, for the third set F7, F8, and F9 where the amount of starch was minimum, the drug release was again much less, since the amount of PVA compared to starch increased. Hence the ratio of starch: PVA has great role to play in the release pattern of the drug and by the choice of proper ratio the release of the drug can be controlled.

### 3.3. Factorial Design Study

The entire factorial design study was based on the drug release profile in pH = 6.8 phosphate buffer medium. The factorial design layout for nine different batches was presented in Table 2. The response surface regression analysis was performed using coded values of factor levels (−1, 0, and +1) for each factor to determine the significance.

**Figure 2:**
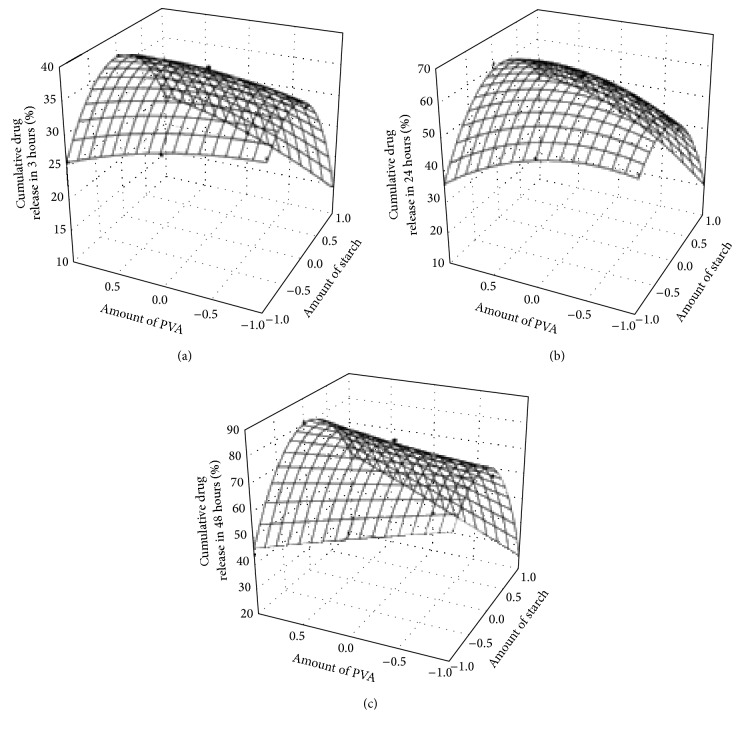
Response surface plots (a) at 180 min, (b) at 1440 min, and (c) at 2880 min.

Table 3 showed the respective *P* values and response surface plots were presented in Figure 2, corresponding to release at 180 minutes (3 hrs), 1440 minutes (24 hrs), and 2880 minutes (48 hrs), respectively. It was observed that, for all nine formulations, the percentage cumulative drug release varied from 13.91% to 34.75% after 180 minutes, after 1440 minutes it varied from 24.27% to 70.98% and after 2880 minutes it varied from 34.11% to 85.97%. The high values of correlation coefficients denoted a good fit. The significance of correlation coefficients was studied using Student's *t*-test. A coefficient was considered significant if the calculated *P* value was less than 0.05, at 95% confidence limit.

Detailed response surface plots were drawn on in vitro drug release experiment data at 180 minutes, 1440 minutes, and 2880 minutes in order to understand contribution of each independent variable over each other (as shown in Tables 4 and 5).

The resultant equations for all three dependent variables *Y*_180_, *Y*_1440_, and *Y*_2880_ in terms of their coded factors were as follows:

(2) Y180=+31.99−4.96X1+1.58X2+4.64X1X2−8.08X12 +1.18X22,Y1440=+71.46−8.80X1−0.01X2+2.89X1X2 −24.14X12−11.66X22,Y2880=+86.11−6.66X1+0.59X2+4.35X1X2 −28.91X12−11.48X22.

A positive value indicated a synergistic effect that favours optimization, while a negative sign represented an antagonistic effect or an inverse effect of the factor on the selected response. It was found that for response *Y*_180_ the contribution of all linear and quadratic terms was significant. The *Y*_1440_ and *Y*_2880_ showed significant quadratic contribution as well as contribution for the amount of starch but less significant contribution for only PVA. Both starch and PVA are polyols, where starch forms the continuous phase with PVA during blending [26]. Thus the contribution of starch was expected to play a significant role in release.

### 3.4. Microscopic Study

Microscopic study showed that all the drug loaded polymer microparticles were round in shape of size within 10 micrometers.

### 3.5. FTIR Study

FTIR spectroscopy was used for observation of any drug polymer interactions. Pure Ornidazole showed a sharp band within 1100 cm^−1^ possibly due to secondary alcoholic group, at 1734.2 cm^−1^ for –C=C– stretching vibration and absorbance at 670 cm^−1^ for monochloro substitution. Starch showed a sharp band spread at 3500–3300 cm^−1^ for carbohydrate alcoholic group and a band at 1161 cm^−1^ for cyclic ether linkages of the sugar backbone. PVA alone showed a band at 3437 cm^−1^ and 1058 cm^−1^ which were possibly due to its bonded secondary alcohol group. The representative formulation of Ornidazole did not reveal any remarkable shift in the sharp peaks of drug and polymers which are mentioned above.

### 3.6. AFM Study

From the AFM study it was found that all the polymer-coated drug particles were of 2.35–2.50 *μ*m size; were spherical in shape; and were having more or less smooth surface. Matrices containing increasing amount of starch-PVA resulted in much improved distribution of the drug over the base polymer matrix.

### 3.7. SEM Study

The SEM study supported the result of AFM and microscopic size analysis.

Thus the results presented a possible effect of the two polymers on delaying the drug release in pH = 6.8 till the first 180 minutes of study as presented in Table 3 with a *P* value <0.0001. Thus the contribution of the two polymers became highly significant at this stage of drug release. The response surface plot presented cumulative drug release with a concave surface, showing maximum drug release with the intermediate amount of both the polymers after which the release decreased for high as well as low amount of polymer. The contour plots also predicted the increase in the amount of PVA facilitating the drug release from the bipolymer matrix which was optimized by changing the ratio of starch: PVA among the designed formulations. F6 was found to be the optimized formulation, since the observed values of drug release for F6 showed significant correlation with the statistically predicted values. The composition of the optimized formulation was starch 53.57% and PVA 44.64%.

**Figure 3:**
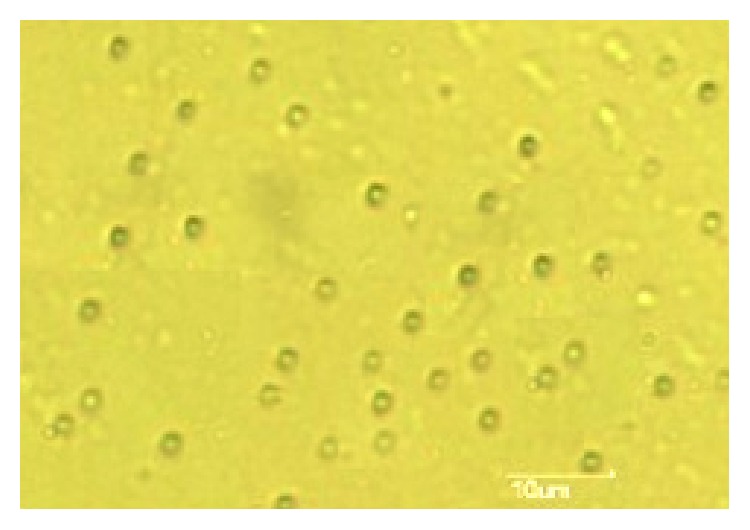
Photomicrograph of drug loaded polymer particle.

**Figure 4:**
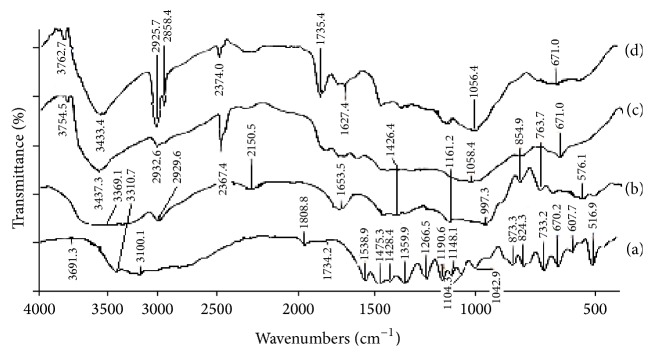
FTIR spectrum of raw materials and microparticles: (a) Ornidazole, (b) starch, (c) PVA, and (d) Ornidazole loaded starch-PVA microparticles.

**Figure 5:**
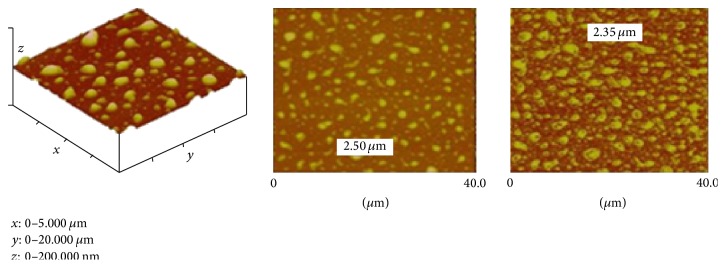
Photomicrographs of polymer-coated drug particles under AFM.

**Figure 6:**
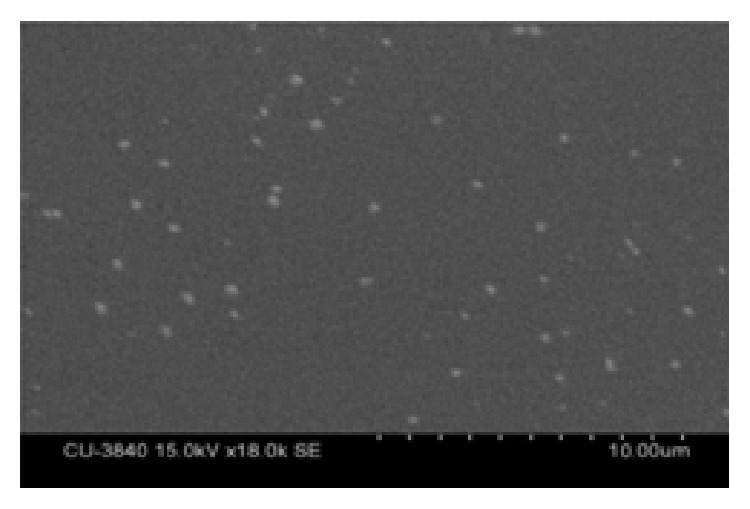
SEM image of polymer-coated drug particle.

The shape and size of the microparticles and their size distribution were of profound importance with regard to the physicochemical properties of the prepared microparticles and thus to the pharmacological action of the drug. Microscopic study, AFM study, and SEM study established the size of the polymer-coated drug loaded microparticles within 2–10 *μ*m range. In FTIR study it appeared that none of the major functional groups of Ornidazole had interaction with the two polymers indicating physical entrapment only. Absence of drug polymer interaction implied that the drug retained its structural integrity within the selected polymer (see Figures 3, 4, 5, and 6). The structural integrity, biological activity, and potency of the drug were therefore expected to remain unaltered [27]. AFM study also revealed that the drug particles were distributed within the base polymer matrix which might be a major determinant for controlled dissolution of Ornidazole from the prepared formulations.

## 4. Conclusion

The use of novel starch-PVA polymer delayed the release of Ornidazole from the spherical microparticles and the extent of release was found to be dependent on the ratio of PVA to starch. Thus we could control the release of the model drug Ornidazole at the preferred site of the GIT by varying the ratio of starch and PVA in the novel polymer.

## Figures and Tables

**Table 1:** Composition of different formulations.

Name of formulation	Amount of starch in mg (*X*_1_)	Amount of PVA in mg (*X*_2_)	Amount of Ornidazole in mg	Amount of glutaraldehyde	Heavy liquid paraffin	Span 20	Concentration H_3_PO_4_
F1	1.5	0.5	2	0.2%	15 mL	2 mL	0.1 mL
F2	1.5	0.8	2	0.2%	15 mL	2 mL	0.1 mL
F3	1.5	1.0	2	0.2%	15 mL	2 mL	0.1 mL
F4	1.2	0.5	2	0.2%	15 mL	2 mL	0.1 mL
F5	1.2	0.8	2	0.2%	15 mL	2 mL	0.1 mL
F6	1.2	1.0	2	0.2%	15 mL	2 mL	0.1 mL
F7	1.0	0.5	2	0.2%	15 mL	2 mL	0.1 mL
F8	1.0	0.8	2	0.2%	15 mL	2 mL	0.1 mL
F9	1.0	1.0	2	0.2%	15 mL	2 mL	0.1 mL

**(a):** 

Batch code	Variable levels in coded forms^b^		*Y*_180_^c^	*Y*_1440_^c^	*Y*_2880_^c^	Percent drug load^d^
	*X*_1_	*X*_2_				%
F7	−1	−1	33.11	47.89	56.09	34.63
F8	−1	0	28.88	56.12	63.97	31.19
F9	−1	1	26.99	41.02	48.57	41.21
F4	0	−1	31.58	58.99	74.11	39.11
F5	0	0	31.99	70.98	85.97	33.97
F6	0	1	34.75	61.09	75.29	33.09
F1	1	−1	13.91	24.27	34.11	39.45
F2	1	0	18.94	39.00	50.57	43.43
F3	1	1	26.34	28.97	43.98	41.33

^b^ *X*_1_ indicated the amount of starch and *X*_2_ the amount of PVA in mg.

^c^ *Y*_*j*_ denoted cumulative drug release at *j*th minutes.

^d^Evaluated on the basis of mass of Ornidazole used at the time of preparation and the mass of Ornidazole found entrapped in each formulation.

**(b):** 

Coded values	Actual values *b*	
	*X*_1_	*X*_2_
−1	1.0	0.5
0	1.2	0.8
1	1.5	1.0

**Table 3:** Analysis of variance.

Responses	Regression	DF	SS	MS	*R* ^2^	*P*
*Y*_180_	FM	5	457.9571	91.5914	0.9983	0.0002
	Residual					
	FM	3	0.7633	0.2544		

*Y*_1440_	FM	5	1317.3516	263.4703	0.9927	0.0021
	Residual					
	FM	3	9.6696	3.2232		

*Y*_2880_	FM	5	2787.2926	557.4585	0.9912	0.0077
	Residual					
	FM	3	49.4445	16.4815		

**Table 4:** Quantitative factor effects and the associated *P* values for all three responses.

Factor	*Y*_180_		*Y*_1440_		*Y*_2880_	
	Factor effects	*P* value	Factor effects	*P* value	Factor effects	*P* value
*X*_1_	−4.6600	0.0002	−4.4553	0.0089	−8.1208	0.0163
*X*_2_	1.1067	0.0126	3.2260	0.0217	2.4325	0.2385
*X*_1_ *X*_1_	−11.1100	<0.0001	−17.6667	0.0008	−28.8458	0.0021
*X*_2_ *X*_2_	−1.3500	0.0323	−6.1126	0.0171	−0.4358	0.889
*X*_1_ *X*_2_	4.1775	0.0005	10.4505	0.0014	13.1488	0.0075

*X*_1_ & *X*_2_ denoted the effect of changing one factor at a time from its lowest to highest level.

*X*_1_ *X*_2_, *X*_1_ *X*_1_, *X*_2_ *X*_2_ are the interaction terms denoted the effect when both the factors were changed simultaneously.

*Y*_180_, *Y*_1440_, *Y*_2880_ denoted cumulative drug release at180, 1440, & 2880 minutes respectively.

**Table 5:** Observed and predicted responses and residual values of optimized formulation (F6).

Response	Formulation		
	Observed	Predicted	Residual
*Y*_180_	36.9500	36.7700	0.1800
*Y*_1440_	61.5082	59.6406	1.8677
*Y*_2880_	80.4700	79.4606	1.0094

*Y*_180_, *Y*_1440_, *Y*_2880_ denoted cumulative drug release at 180, 1440, & 2880 minutes respectively.

## References

[1] Florence A. T., Jani P. U., Rolland A. (1993). Particulate delivery: the challenge of the oral route. *Pharmaceutical Particulate Carriers: Therapeutic Applications*.

[2] Chen H., Langer R. (1998). Oral particulate delivery: status and future trends. *Advanced Drug Delivery Reviews*.

[3] Uhrich K. E., Cannizzaro S. M., Langer R. S., Shakesheff K. M. (1999). Polymeric systems for controlled drug release. *Chemical Reviews*.

[4] Greenhalgh K., Turos E. (2009). In vivo studies of polyacrylate nanoparticle emulsions for topical and systemic applications. *Nanomedicine: Nanotechnology, Biology, and Medicine*.

[5] Bhardwaj T. R., Kanwar M., Lal R., Gupta A. (2000). Natural gums and modified natural gums as sustained-release carriers. *Drug Development and Industrial Pharmacy*.

[6] Verhoeven E., Vervaet C., Remon J. P. (2006). Xanthan gum to tailor drug release of sustained-release ethylcellulose mini-matrices prepared via hot-melt extrusion: in vitro and in vivo evaluation. *European Journal of Pharmaceutics and Biopharmaceutics*.

[7] George M., Abraham T. E. (2007). pH sensitive alginate-guar gum hydrogel for the controlled delivery of protein drugs. *International Journal of Pharmaceutics*.

[8] Chang C.-P., Leung T.-K., Lin S.-M., Hsu C.-C. (2006). Release properties on gelatin-gum arabic microcapsules containing camphor oil with added polystyrene. *Colloids and Surfaces B: Biointerfaces*.

[9] Agnihotri S. A., Jawalkar S. S., Aminabhavi T. M. (2006). Controlled release of cephalexin through gellan gum beads: Effect of formulation parameters on entrapment efficiency, size, and drug release. *European Journal of Pharmaceutics and Biopharmaceutics*.

[10] Matricardi P., Cencetti C., Ria R., Alhaique F., Coviello T. (2009). Preparation and characterization of novel Gellan gum hydrogels suitable for modified drug release. *Molecules*.

[11] Tarvainen M., Peltonen S., Mikkonen H. (2004). Aqueous starch acetate dispersion as a novel coating material for controlled release products. *Journal of Controlled Release*.

[12] Mano J. F., Koniarova D., Reis R. L. (2003). Thermal properties of thermoplastic starch/synthetic polymer blends with potential biomedical applicability. *Journal of Materials Science: Materials in Medicine*.

[13] Park E. H., George E. R., Muldon M. A., Flammino A. (1994). Thermoplastic starch blends with poly(vinyl alcohol): processability, physical properties, and Biodegradability. *Polymer News*.

[14] Lawton J. W. (1996). Effect of starch type on the properties of starch containing films. *Carbohydrate Polymers*.

[15] Simmons S., Thomas E. L. (1995). Structural characteristics of biodegradable thermoplastic starch/poly(ethylene-vinyl alcohol) blends. *Journal of Applied Polymer Science*.

[16] Kennedy J. F., Knill C. J. (1996). Polymers from agricultural coproducts. Acs symposium series no. 575. Edited by M. L. Fishman, R. B. Friedman and S. J. Huang. American chemical society, Washington DC, 1994. pp. viii + 247, price US$69.95. ISBN 0-8412-3041-2. *Polymer International*.

[17] Tudorachi N., Cascaval C. N., Rusu M., Pruteanu M. (2000). Testing of polyvinyl alcohol and starch mixtures as biodegradable polymeric materials. *Polymer Testing*.

[18] Huang S. J., Ho L. H., Huang M. T., Koenig M. F., Cameron J. A., Doi Y., Fukuda K. (1994). Similarities and differences between biodegradation and non-enzymatic degradation. *Biodegradable Plastics and Polymers*.

[19] Cavalcanti O. A., Van den Mooter G., Caramico-Soares I., Kinget R. (2002). Polysaccharides as excipients for colon-specific coatings. Permeability and swelling properties of casted films. *Drug Development and Industrial Pharmacy*.

[20] Ramachandran S., Nandhakumar S., Dhanaraju M. D. (2011). Formulation and characterization of glutaraldehyde cross-linked chitosan biodegradable microspheres loaded with famotidine. *Tropical Journal of Pharmaceutical Research*.

[21] Semalty A., Aswal R. (2014). Chitosan microspheres of metformin hydrochloride and the effect of using different concentrations of crosslinking agent. *International Research Journal for Inventions in Pharmaceutical Sciences*.

[22] Manjanna K. M., Rajesh K. S., Shivakumar B. (2013). Formulation and optimization of natural polysaccharide hydrogel microbeads of aceclofenac sodium for oral controlled drug delivery. *The American Journal of Medical Sciences and Medicine*.

[23] Petrovic A., Ibric S., Trajkovic S., Popovic R., Djuric Z., Popadic D. (2009). An investigation into effects of *in vitro* Test condition on the release properties of theophylline from HPMC matrices using factorial design. *Archives of Pharmacal Research*.

[24] Malafaya P. B., Stappers F., Reis R. L. (2006). Starch-based microspheres produced by emulsion crosslinking with a potential media dependent responsive behavior to be used as drug delivery carriers. *Journal of Materials Science: Materials in Medicine*.

[25] Patel P., Roy A., Vinod Kumar S. M., Kulkarni M. (2011). Formulation and evaluation of colon targeted tablets of Ornidazole for the treatment of amoebiasis. *International Journal of Drug Development and Research*.

[26] Lu D. R., Xiao C. M., Xu S. J. (2009). Starch-based completely biodegradable polymer materials. *Express Polymer Letters*.

[27] Khan M. S., Vishakante G. D., Bathool A., Kumar R. (2012). Preparation and evaluation of spray dried microparticles using chitosan and novel chitosan derivative for controlled relea se of an antipsychotic drug. *International Journal of Biological & Pharmaceutical Research*.

